# Supplementary material for: Carbon and microbes in a degrading palsa mire are distinct from a peatland and a wider connected sub-Arctic fluvial system
Source: ISME Commun. 2025 Dec 18;5(1):ycaf240. doi: 10.1093/ismeco/ycaf240 (PMC12766748; doi:10.1093/ismeco/ycaf240)
Supplement: 20251230_Supplementary_ycaf240 [file 20251230_supplementary_ycaf240.pdf]

ISME Communications

Carbon and microbes in a degrading palsa mire are distinct from a peatland and a wider connected sub-Arctic fluvial system

Supplementary

Tuomela, Nea[1*]; Elovaara, Samu[2]; Hultman, Jenni[3]; Kaartokallio, Hermanni[2]; Thomas, David N.[1]

[1] Faculty of Biological and Environmental Sciences, University of Helsinki, Helsinki, Finland

[2] Finnish Environment Institute (SYKE), Helsinki, Finland

[3] Natural Resources Institute Finland (LUKE), Helsinki, Finland

*Corresponding author, nea.tuomela@helsinki.fi, Faculty of Biological and Environmental Sciences, University of Helsinki, Viikinkaari 1, 00790 Helsinki, Finland

The following document contains supplementary material for "Carbon and microbes in a degrading palsa mire are distinct from peatland and a wider connected sub-Arctic fluvial system".

14 Pages, 12 Figures, 1 Table

Contents

List of Figures 2
Supplementary Methods 3
Supplementary Methods 1: Cell Abundance 3
Supplementary Figures 3
Supplementary Tables 14

List of Figures

Supplementary Figure 1. Examples of the sampled soil and water sites in palsa mire (left), and peatland site (right). In palsa site permafrost degradation was visible with collapse features.

Supplementary Figure 2. Bacterial community composition in phylum level based 16S rRNA gene annotations from metagenomes divided by samples site (a) palsa, (b) peat and (c) streams sites.

Supplementary Figure 3. Bacterial community composition in order level based 16S rRNA gene annotations from metagenomes divided by samples site (a) palsa, (b) peat and (c) streams sites.

Supplementary Figure 4. Archaeal community composition in order level based 16S rRNA gene annotations from metagenomes divided by samples site (a) palsa, (b) peat and (c) streams sites.

Supplementary Figure 5. Gating examples used in to derive data for bacterial cell number data for (a) high nucleic acid and (b) low nucleic acid cells.

Supplementary Figure 6. Screeplot used to justify the selection of principal components with the biogeochemical data.

Supplementary Figure 7. Shannon diversity index of the sampled soil and water sites.

Supplementary Figure 8. Microbial respiration rate (a) and high nucleic acid bacterial cell numbers (b,c) during degradation experiment

Supplementary Figure 9. CDOM absorption coefficients at selected wavelengths in the peat, Kidisjoki River and Utsjoki River sites (a) and palsa sites (b) during the degradation experiment.

Supplementary Figure 10. CDOM spectral slopes at selected wavelength ranges and the S_{ratio} in the peat, Kidisjoki River and Utsjoki River sites (a) and palsa sites (b) during the degradation experiment.

Supplementary Figure 11. Coble's peaks A, C, M and T of FDOM and the biological index, the humification index and the fluorescence index in the peat, Kidisjoki River and Utsjoki River sites (a) and palsa sites (b) during the degradation experiment.

Supplementary Figure 12. Nutrient concentrations in the peat, Kidisjoki River and Utsjoki River sites (a) and palsa sites (b) during the degradation experiment.

Supplementary Methods

Supplementary Methods 1: Cell Abundance

Bacterial cell number was analyzed using flow cytometry with LSRFortessa (BD Biosciences) at University of Helsinki Flow Cytometry Unit. Samples were fixed with paraformaldehyde (concentration 0.9%), incubated for 15 minutes in the dark and stored in -80 °C until analysis. Prior to analysis samples were thawed, filtered with strain cap tubes (pore size 35 µm, Fisher Scientific), stained with Sybr Green I nucleic acid stain (final concentration 1:10 000 Vol.) and incubated for 10 minutes in dark and room temperature. Each sample was measured in triplicate and controls with ultrapure water were run after every 10 measurements. Palsa pond samples were diluted in 1:10 ratio with ultrapure water (MQ). CountBright™ Absolute Counting Beads (Thermo Fisher Scientific) were used to calculate the concentration of cells per millilitre. Bacteria were detected and counted by plotting green fluorescence against side scatter (Supplementary Figure 5) with FlowJo software v.10.10.0 (BD Biosciences).

Supplementary Figures

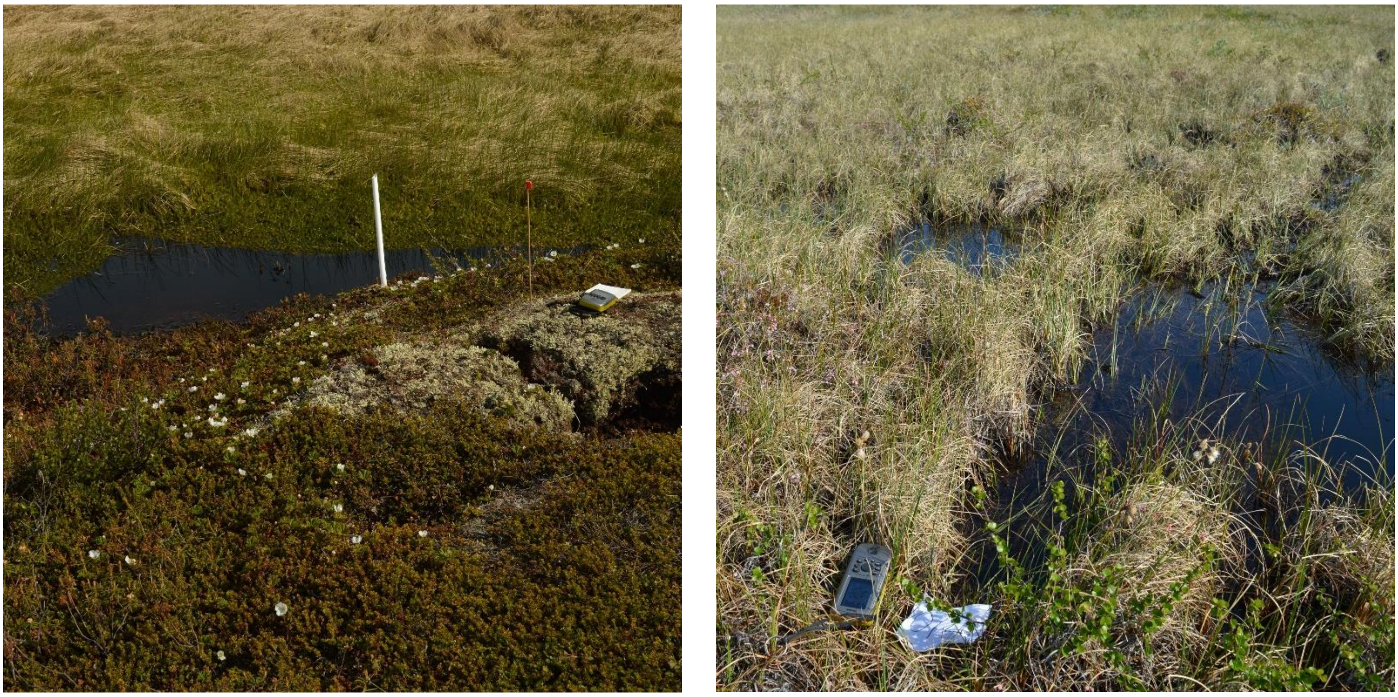

Supplementary Figure 1. Examples of the sampled soil and water sites in palsa mire (left), and peatland site (right). In palsa site permafrost degradation was visible with collapse features.

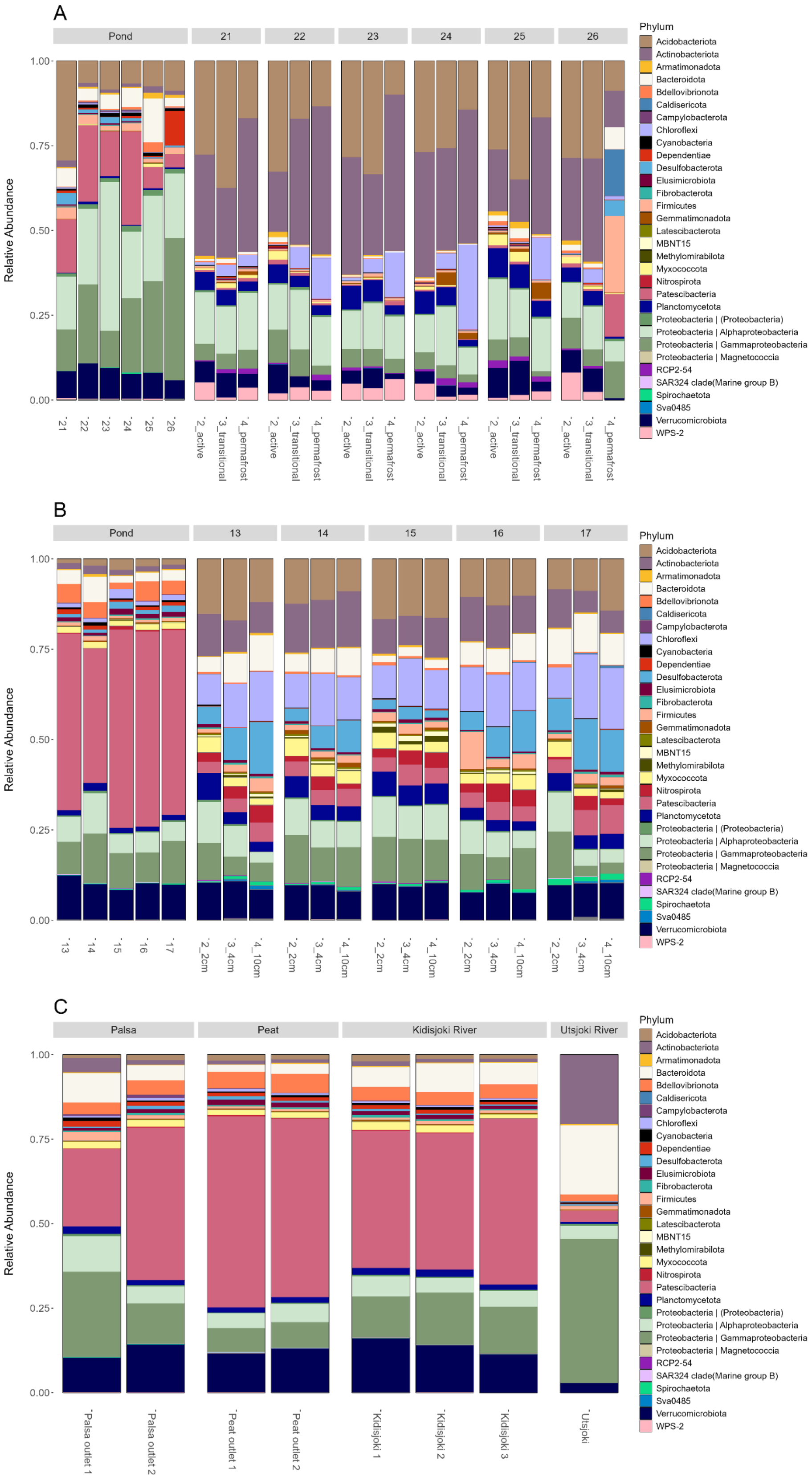

Supplementary Figure 2. Bacterial community composition in phylum level for each individual sample in (a) palsa site, (b) peat site and (c) stream sites. For palsa soils, active layer refers to the dry peat layer (20-25cm below ground), transitional to the depth of freezing in the soil (23-33cm) and permafrost the frozen soil (49-59cm). In stream sites outlet 1 is left bar and outlet 2 is the right one. Relative abundance lower than 0.01% are filtered out.

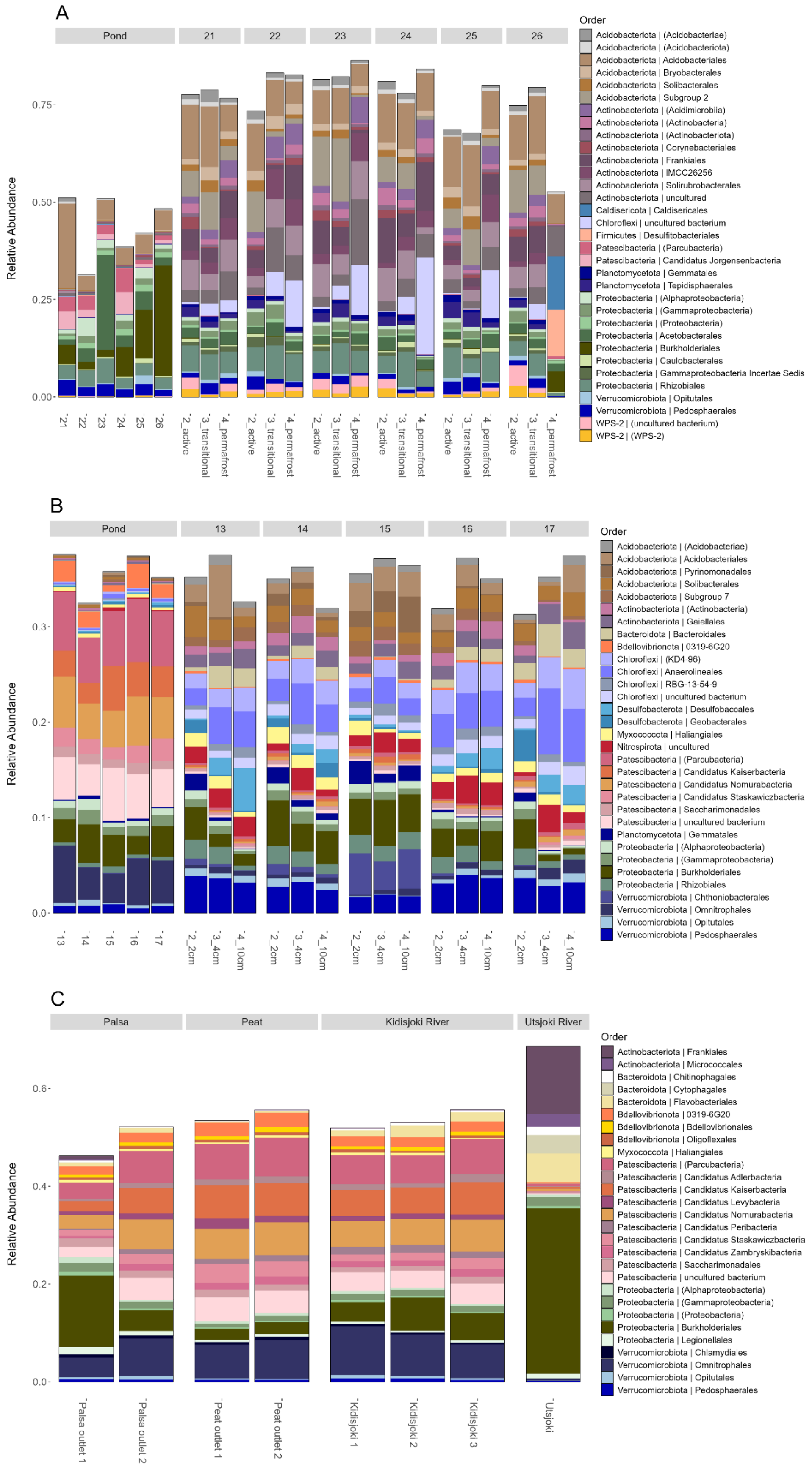

Supplementary Figure 3. Bacterial community composition in order level for each individual samples (a) palsa site, (b) peat site and (c) stream sites. In stream sites outlet 1 is left bar and outlet 2 is the right one. Relative abundance lower than 0.38% are filtered out.

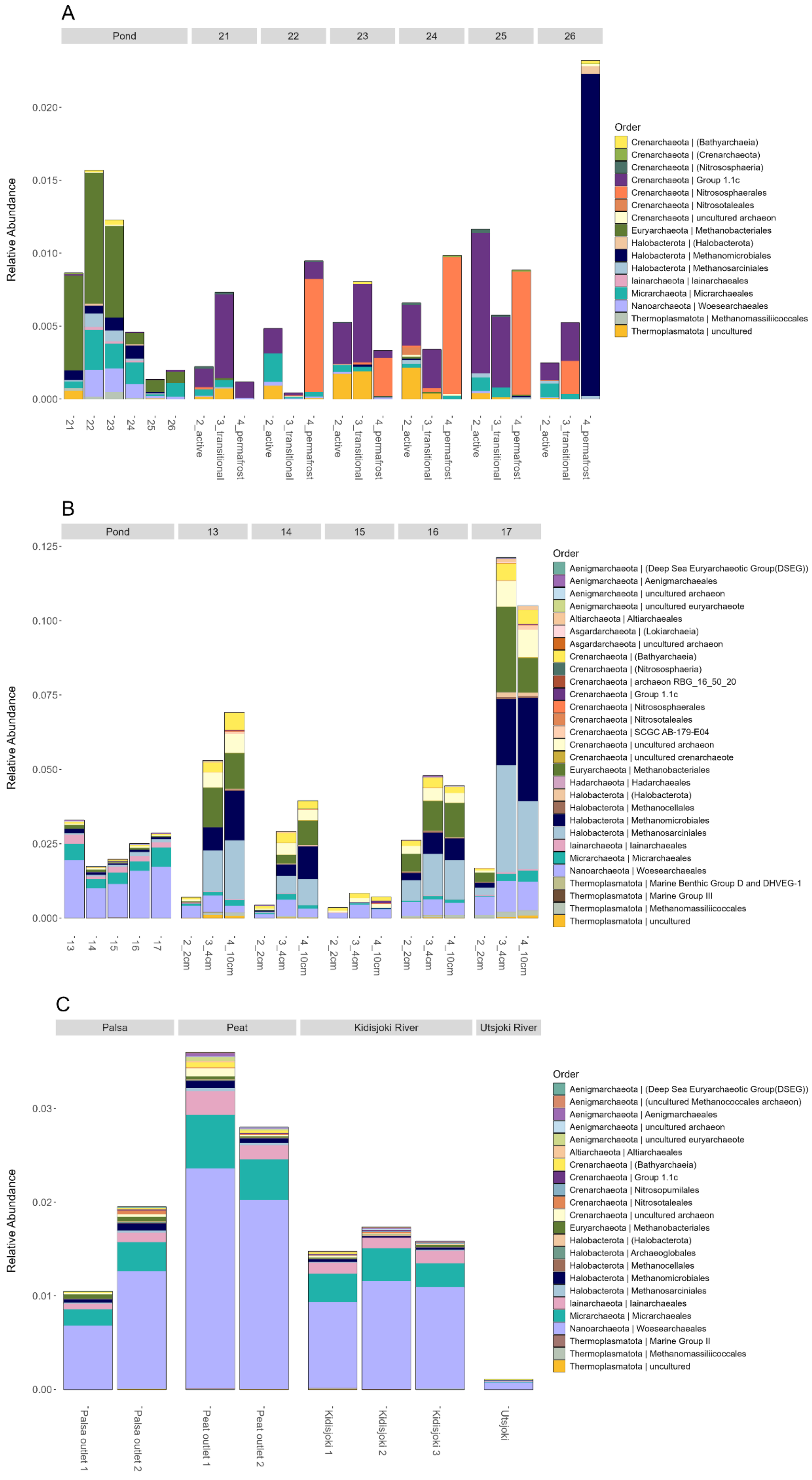

Supplementary Figure 4. Archaeal communities in order level for each sample in (a) palsa site, (b) peat site and (c) stream sites. In stream sites outlet 1 is left bar and outlet 2 is the right one.

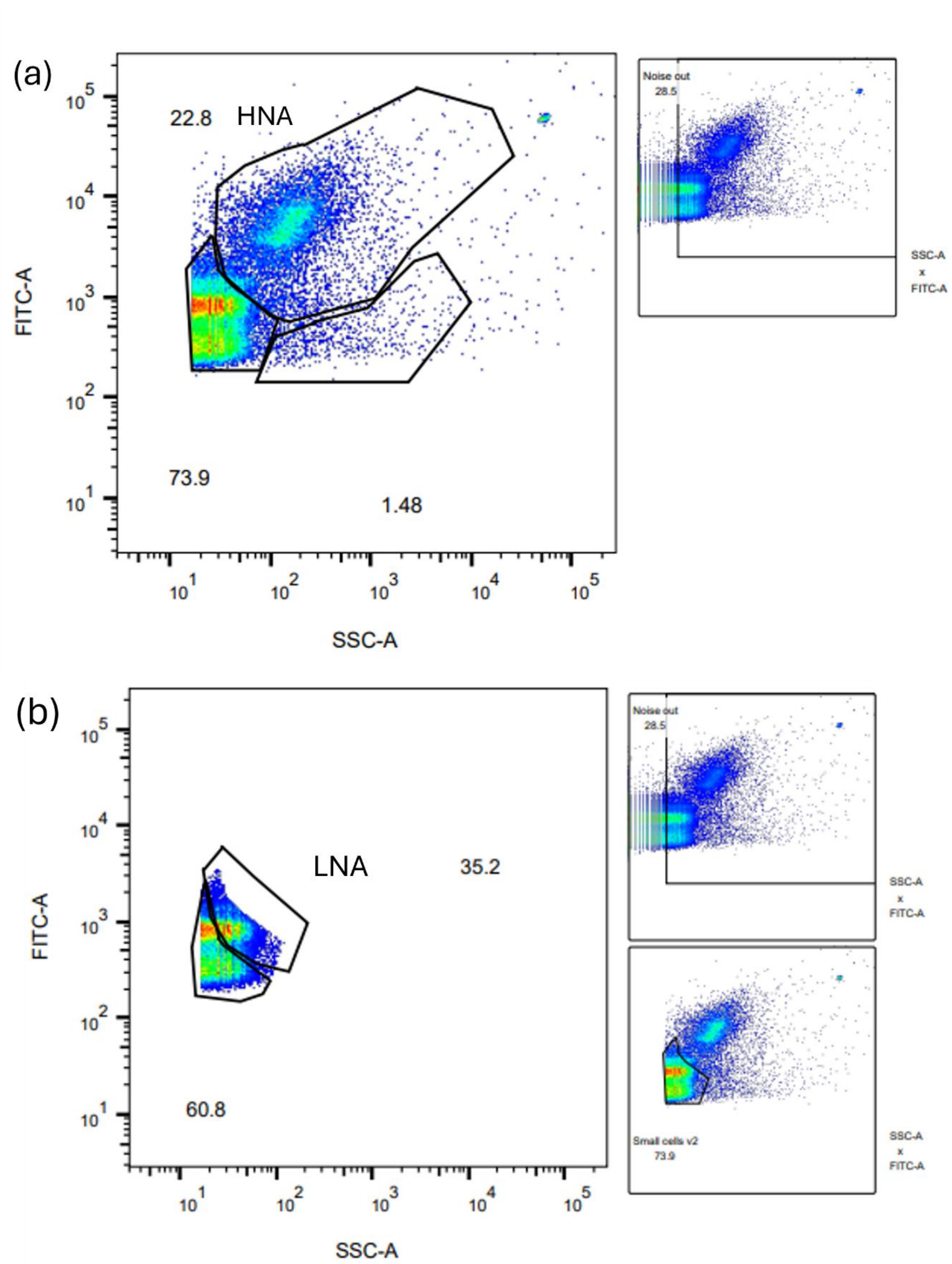

Supplementary Figure 5. Examples of the gating approach used for cell enumeration of (a) HNA cells and (b) LNA in diluted palsa pond samples

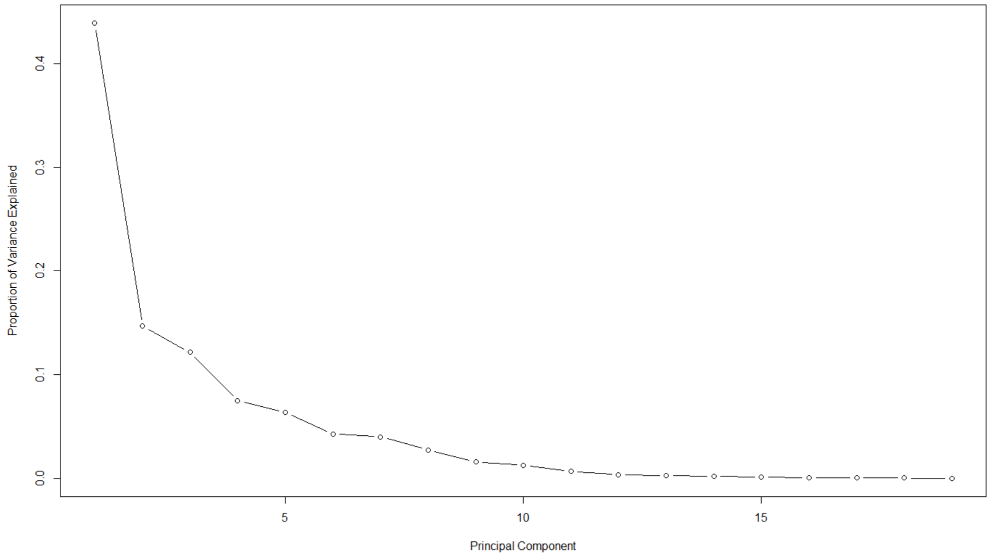

Supplementary Figure 6. Screeplot was used to justify the selection of principal components with the biogeochemical data.

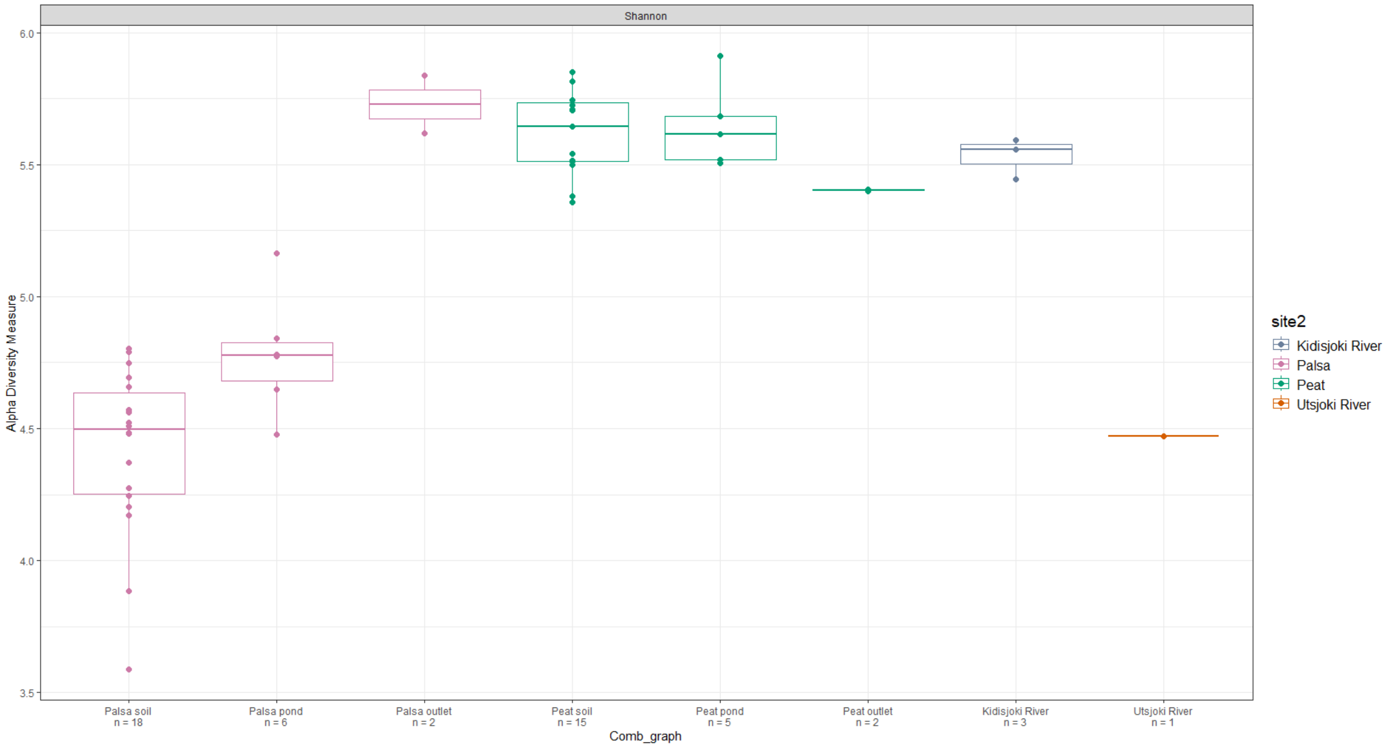

Supplementary Figure 7. Shannon diversity index of the 16S rRNA gene annotations from metagenomes. Total number of samples indicated in x-axis below the sample name.

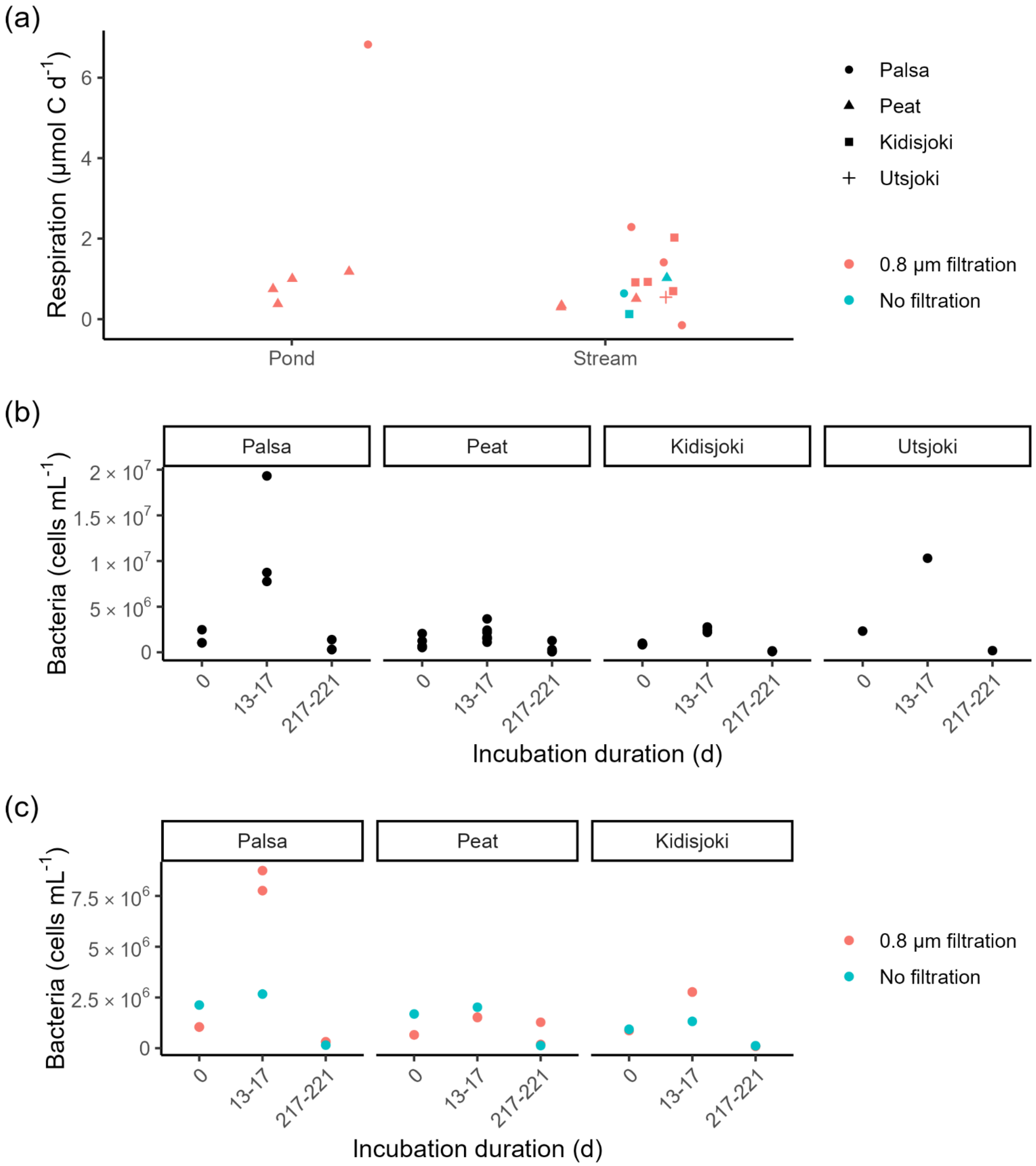

Supplementary Figure 8: Microbial respiration rate (µmol C L^{1-} d^{-1}) during the first 13-17 days of the degradation experiment (a). Abundance of HNA bacteria in all filtered degradation experiment samples except the palsa pond, which was removed because of much higher HNA abundance (b). Abundance of HNA bacteria in the stream sites where the non-filtered full community controls where collected (c).

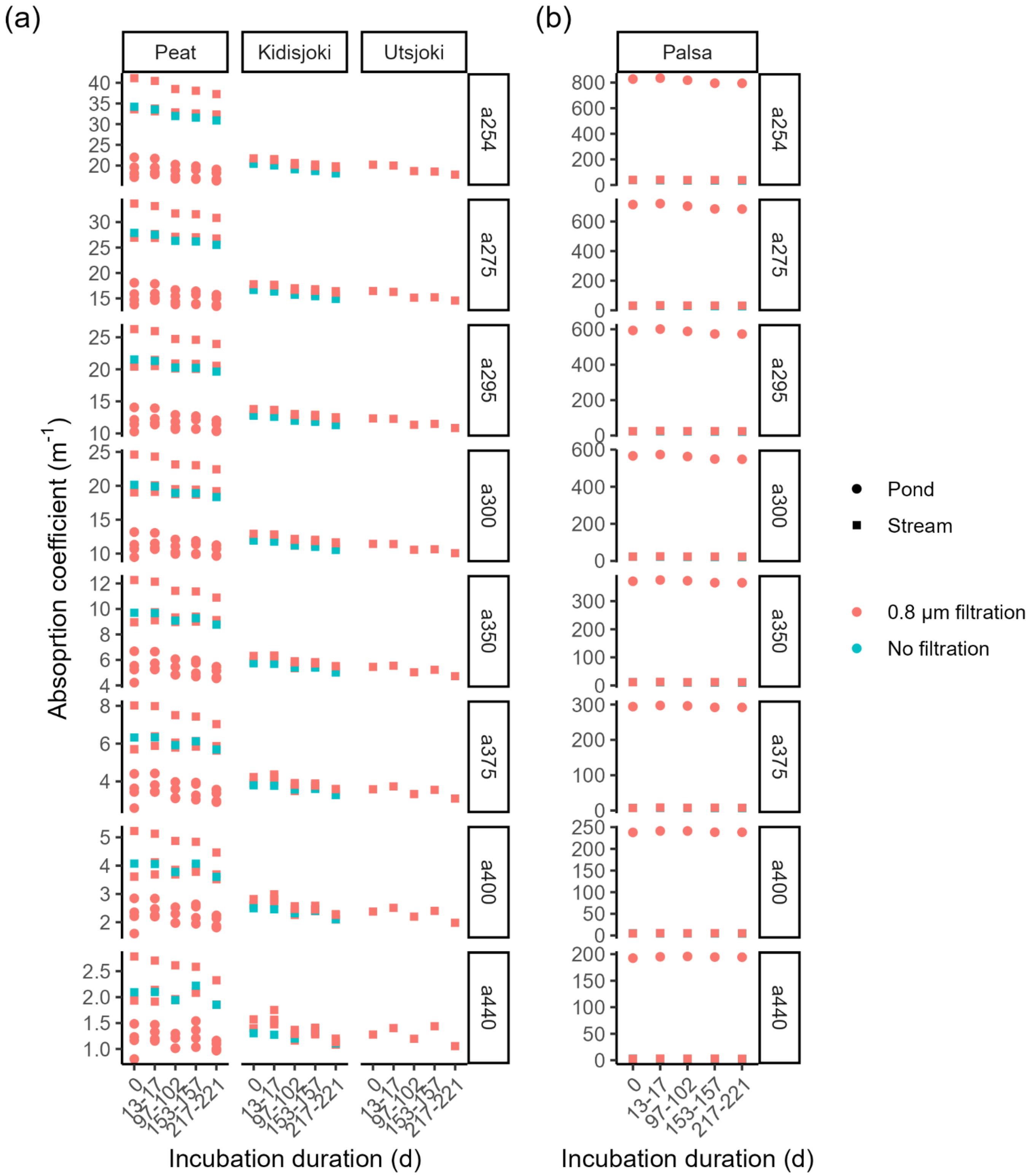

Supplementary Figure 9: CDOM absorption coefficients at selected wavelengths in the peat, Kidisjoki River and Utsjoki River sites (a) and palsa sites (b) during the degradation experiment.

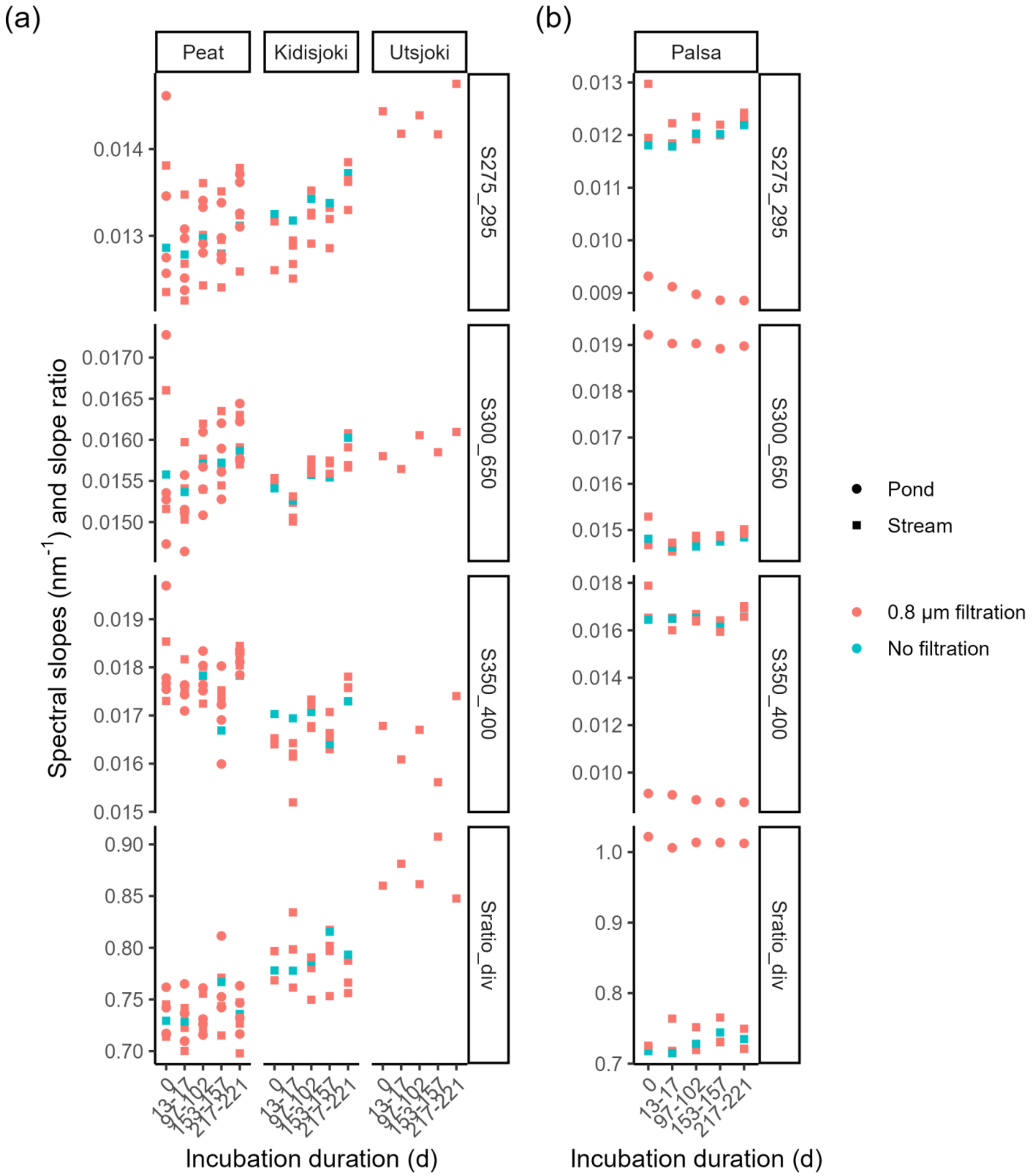

Supplementary Figure 10: CDOM spectral slopes at selected wavelength ranges and the S_{ratio} in the peat, Kidisjoki River and Utsjoki River sites (a) and palsa sites (b) during the degradation experiment.

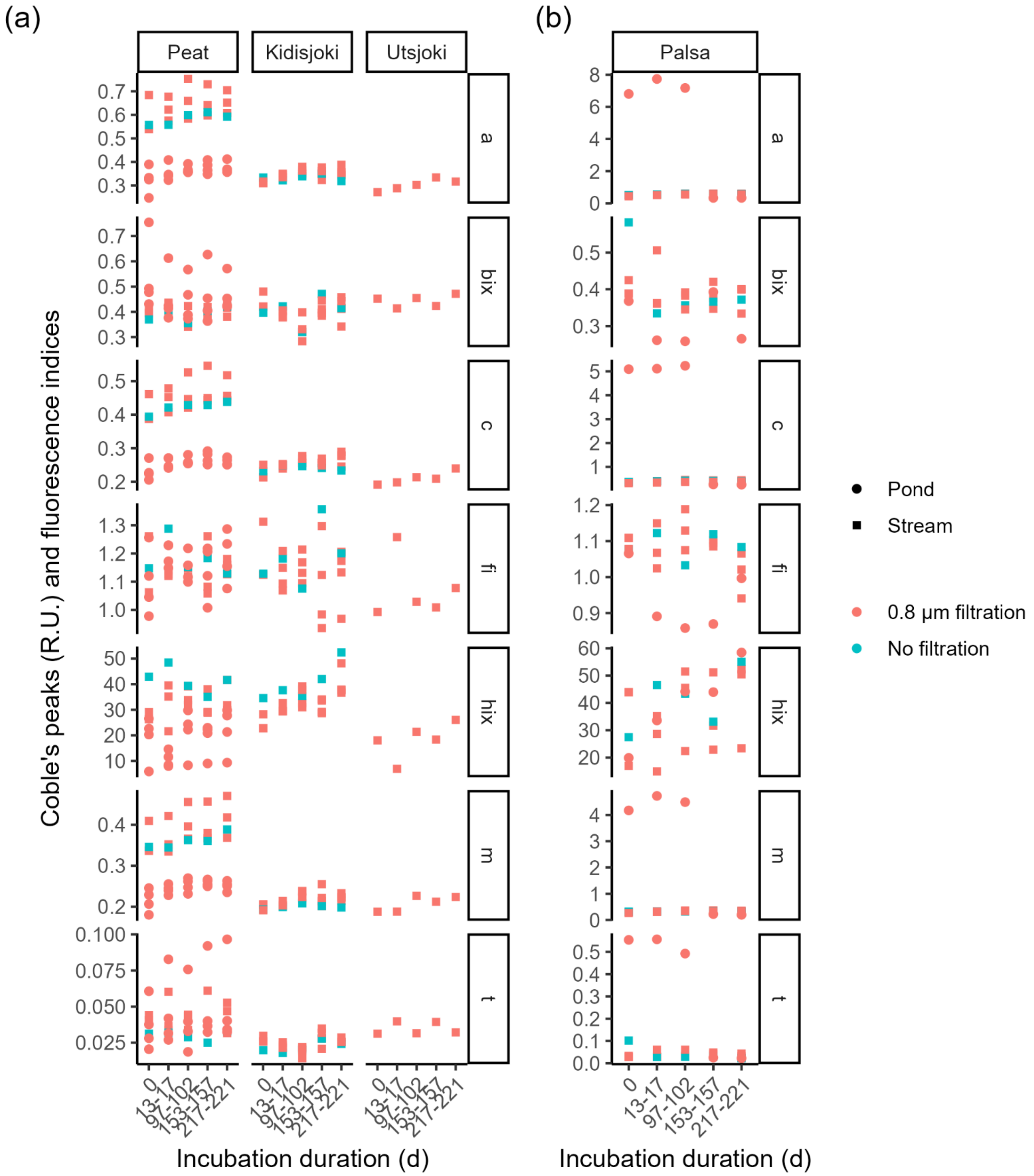

Supplementary Figure 11: Coble's peaks A, C, M and T of FDOM and the biological index (bix), the humification index (hix) and the fluorescence index (fi) in the peat, Kidisjoki River and Utsjoki River sites (a) and palsa sites (b) during the degradation experiment.

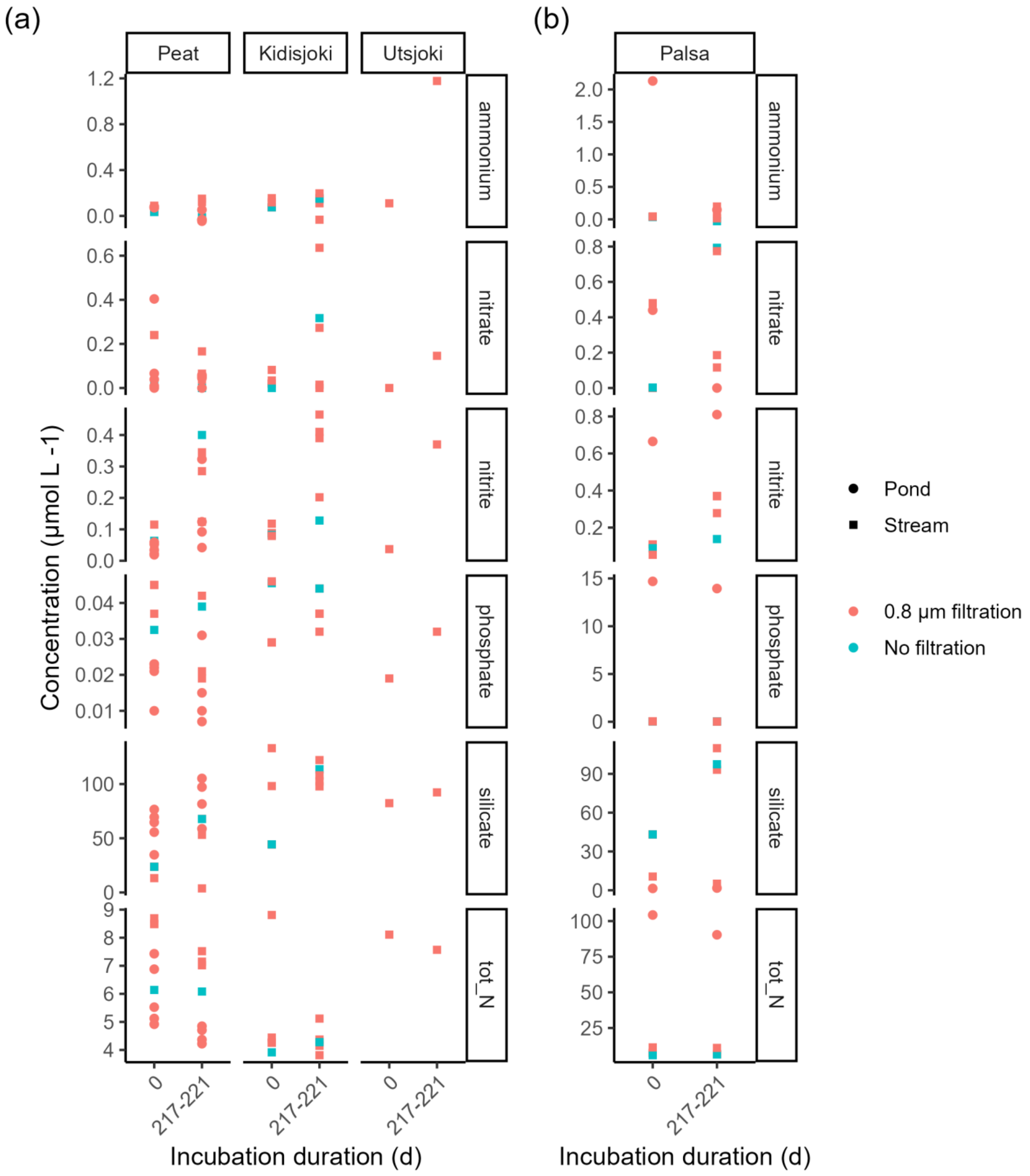

Supplementary Figure 12: Nutrient concentrations in the peat, Kidisjoki River and Utsjoki River sites (a) and palsa sites (b) during the degradation experiment.

Supplementary Tables

Supplementary Table 1. Filtered water volumes for the microbial community composition. Each sample was filtered sequentially with 0.45μm and 0.1μm pore sized filters. DNA extraction was performed individually and extracted DNA was combined with same ratio as the filtered volumes prior to sequencing.

Sample	0.45 μm (ml)	0.1 μm (ml)
1	1000	500
4	1060	500
5	1400	616
10	560	500
13	630	500
14	387	387
15	470	282
16	366	366
17	262	262
18	500	498
21	66	66
22	64	64
23	40	40
24	36	36
25	28	26
26	130	84
27	398	402
28	340	398
30	500	330
